# Dimethyl 4,4′-dihy­droxy-3,3′-{[(3a*RS*,7a*RS*)-2,3,3a,4,5,6,7,7a-octa­hydro-1*H*-1,3-benzimidazole-1,3-di­yl]bis­(methyl­ene)}dibenzoate

**DOI:** 10.1107/S1600536811040906

**Published:** 2011-10-12

**Authors:** Augusto Rivera, Diego Quiroga, Jaime Ríos-Motta, Karla Fejfarová, Michal Dušek

**Affiliations:** a Departamento de Química, Universidad Nacional de Colombia, Ciudad, Universitaria, Bogotá, Colombia; b Institute of Physics ASCR, v.v.i., Na Slovance 2, 182 21 Praha 8, Czech Republic

## Abstract

The title compound, C_25_H_30_N_2_O_6_, has the imidazolidine ring in an envelope conformation. There are two intra­molecular O—H⋯N hydrogen-bond inter­actions with graph-set motif *S*(6). The cyclo­hexane ring adopts a slightly distorted chair conformation. One methyl carboxyl­ate substituent forms a dihedral angle of 12.00 (5)° with the plane of the benzene ring, while the other methyl carboxyl­ate group is almost coplanar, making a dihedral angle of 2.26 (9)°. In the crystal, pairs of inter­molecular C—H⋯O hydrogen bonds form racemic dimers, corresponding to an *R*_2_^2^(18) graph-set motif. Further weak C—H⋯O inter­actions generate a chain running along the *c* axis.

## Related literature

For related structures, see: Rivera *et al.* (2011**a* ▶,*b* ▶,c* ▶). For the synthesis of the precursor, see: Murray-Rust & Riddell (1975 ▶). For puckering parameters, see: Cremer & Pople (1975 ▶). For hydrogen-bond graph-set nomenclature, see: Bernstein *et al.* (1995 ▶).
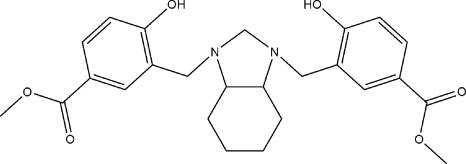


## Experimental

### 

#### Crystal data

C_25_H_30_N_2_O_6_
*M* *_r_* = 454.5
Monoclinic, 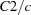

*a* = 26.3472 (6) Å
*b* = 9.1432 (1) Å
*c* = 21.6585 (4) Å
β = 121.139 (3)°
*V* = 4465.7 (2) Å^3^
*Z* = 8
Cu *K*α radiation
μ = 0.80 mm^−1^
*T* = 120 K
0.41 × 0.23 × 0.16 mm

#### Data collection

Agilent Xcalibur diffractometer with an Atlas (Gemini ultra Cu) detector
Absorption correction: multi-scan (*CrysAlis PRO*; Agilent, 2010 ▶) *T*_min_ = 0.853, *T*_max_ = 1
17421 measured reflections
3970 independent reflections
3309 reflections with *I* > 3σ(*I*)
*R*_int_ = 0.028

#### Refinement

*R*[*F* ^2^ > 2σ(*F* ^2^)] = 0.035
*wR*(*F* ^2^) = 0.097
*S* = 1.54
3970 reflections
304 parameters
H atoms treated by a mixture of independent and constrained refinement
Δρ_max_ = 0.26 e Å^−3^
Δρ_min_ = −0.18 e Å^−3^

### 

Data collection: *CrysAlis PRO* (Agilent, 2010 ▶); cell refinement: *CrysAlis PRO*; data reduction: *CrysAlis PRO*; program(s) used to solve structure: *SIR2002* (Burla *et al.*, 2003 ▶); program(s) used to refine structure: *JANA2006* (Petříček *et al.*, 2006 ▶); molecular graphics: *DIAMOND* (Brandenburg & Putz, 2005 ▶); software used to prepare material for publication: *JANA2006*.

## Supplementary Material

Crystal structure: contains datablock(s) global, I. DOI: 10.1107/S1600536811040906/bt5658sup1.cif

Structure factors: contains datablock(s) I. DOI: 10.1107/S1600536811040906/bt5658Isup2.hkl

Supplementary material file. DOI: 10.1107/S1600536811040906/bt5658Isup3.cml

Additional supplementary materials:  crystallographic information; 3D view; checkCIF report

## Figures and Tables

**Table 1:** Hydrogen-bond geometry (Å, °)

*D*—H⋯*A*	*D*—H	H⋯*A*	*D*⋯*A*	*D*—H⋯*A*
O3—H3⋯N1	0.90 (2)	1.80 (2)	2.6383 (18)	154.3 (16)
O6—H6⋯N2	0.87 (2)	1.88 (2)	2.6814 (18)	153.0 (18)
C2—H2⋯O1^i^	0.96	2.57	3.414 (2)	146
C4—H4*b*⋯O1^i^	0.96	2.58	3.353 (2)	137
C16—H16*c*⋯O6^ii^	0.96	2.59	3.350 (2)	136

Symmetry codes: (i) 
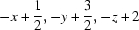
; (ii) 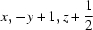.

**Fig. 1.**
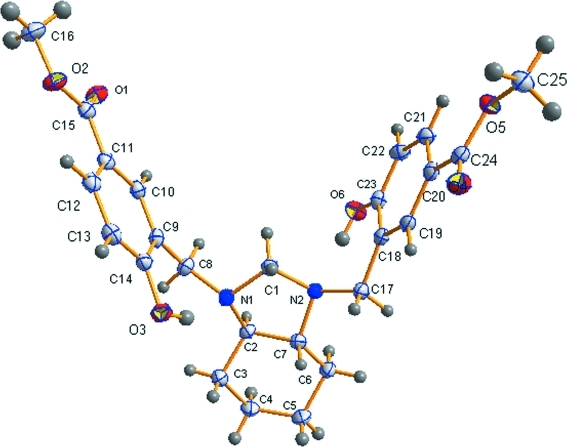
A view of (I) with the numbering scheme. Displacement ellipsoids are drawn at the 50% probability level.

**Fig. 2.**
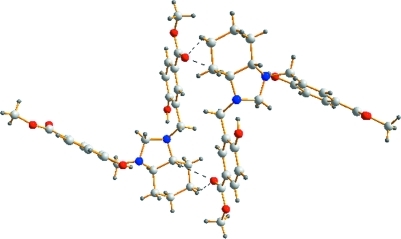
Dimer formation of the title compound by a R22(18) ring motif.

**Fig. 3.**
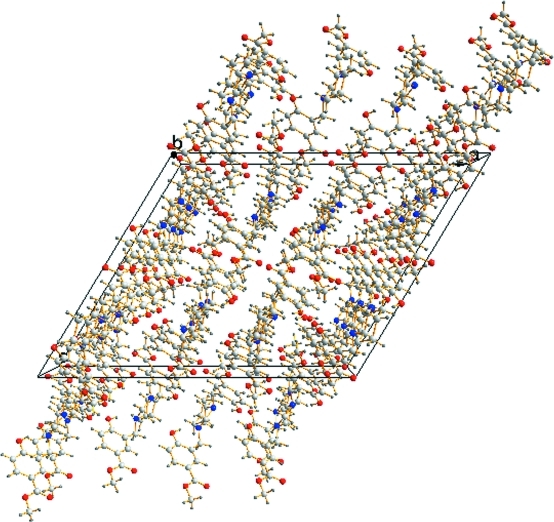
Packing of the molecules of the title compound view along the c axis.

## supplementary crystallographic information

### Comment

The synthesis of the title compound (I) represents an expansion of our previous work exploring the substituent effects on the solid state structures of *di-*Mannich bases (Rivera *et al.*, 2011**a*,*b*,c*). In the title compound (I), C_25_H_30_N_2_O_6_, the heterocyclc ring has a envelope conformation on C7 (Q(2) = 0.4439 (15) Å, φ = 296.59 (19)°). It is surprising, however, that of the 12 fused 1,3-disubstituted-(3a*R*,7a*R*/3a*S*,7a*S*)-2,3,3a,4,5,6,7,7a-octahydro-1*H*-1,3-benzimidazole of this type studied to date by us, including the title compound, only two has an envelope conformation on aminalic carbon (NCH_2_N); the other all have a twisted conformation on C—C bond joint to both rings. The molecular conformation is stabilized by two intramolecular O—H···N hydrogen-bond interaction with set graph motif S(6) (Bernstein,*et al.* 1995). The cyclohexane ring adopt a slightly distorted chair conformation (Cremer & Pople, 1975) with puckering parameters Q, θ and φ of 0.5834 (16) Å, 5.35 (16)°, 300.8 (17)°. In the molecule of the title compound (Fig. 1), bond lengths and angles are generally within normal ranges and comparable with those observed in related compounds (Rivera *et al.*, 2011**a*,*b*,c*). Whereas the first carbonyl group is coplanar with the phenyl ring [torsion angle C10—C11—C15—O1 of 1.3 (2)°], the second carbonyl group is slightly twisted out of the plane of the aromatic ring. The torsion angle C19—C20—C24—O4 amounts to -10.1 (2)°. Therefore, the differences in orientation likely arise as a result of steric considerations with respect to the crystal packing.

The crystal packing is dominated by non-conventional C—H···O hydrogen bond interactions, Table 1. In the racemic crystal of title compound, a pair of the enantiomers are bonded together with two intermolecular bifurcate hydrogen bonds (Figure 2), generating a *R*_2_^2^(18) graph*-*set motifs (Bernstein,*et al.* 1995). So, O1 can form two bifurcate intermolecular hydrogen bonds with two hydrogen atoms, C2—H2···O1 [C···O = 3.414 (2) Å] and C4—H4b···O1 [C···O = 3.353 (2) Å], but the two hydrogen atoms are in the same molecule, which indicates that one molecule in the crystals can be connected with one neighboring molecule by two intermolecular C—H···O bifurcate hydrogen bonds. The racemic dimers are further connected by additional C16—H16*c*···O6 hydrogen bonds, forming a supramolecular chain along the *c* axis (Figure 3). Thus, the ability of O6—H6 hydroxyl group to act as a hydrogen bridge donor and acceptor is important for the formation of crystals packing of the title compound. However, this additional H-bonding does not influence the intramolecular O—H···N distance, which shows a typical O···N distances of 2.6814 (18) Å (Rivera *et al.*, 2011**a*,*b*,c*).

### Experimental

A solution of methyl 4-hydroxybenzoate (304 mg, 2.00 mmol) in dioxane (3 ml) was added dropwise to (2*R*,7*R*,11*S*,16S)-1,8,10,17- tetraazapentacyclo[8.8.1.1^8,17^.0^2,7^.0^11,16^]icosane (276 mg, 1.00 mmol) in dioxane (3 ml) and water (4 ml), prepared following previously described procedures (Murray-Rust & Riddell, 1975). The mixture was refluxed for about 10 h. The solvent was evaporated under reduced pressure until a sticky residue appeared. The product was purified by chromatography on a silica column, and subjected to gradient elution with benzene:ethyl acetate (yield 20%, m.p. = 449–450 K). Single crystals of racemic (I) were grown from a chloroform: methanol solution by slow evaporation of the solvent at room temperature over a period of about 2 weeks.

^1^H NMR (CDCl_3_, 400 MHz): δ 1.30 (4*H*, m), 1.86 (2*H*, m), 2.06 (2*H*, m), 2.38 (2*H*, m), 3.53 (2*H*, s, NCH_2_N), 3.54 (2*H*, *d*, ^2^*J*_H,H_ = 14.0 Hz, ArCH_2_N), 4.20 (2*H*, *d*, ^2^*J*_H,H_ = 14.0 Hz, ArCH_2_N), 3.84 (6*H*, s, CH_3_), 6.83 (2*H*, *d*, ^3^*J*_H,H_ = 8.4 Hz), 7.68 (2*H*, s), 7.86 (2*H*, *dd*, ^4^*J*_H,H_ = 1.6 Hz, ^3^J_H,H_ = 8.4 Hz). ^13^C NMR (CDCl_3_, 100 MHz): δ 23.9, 28.8, 51.8, 56.0, 69.1, 75.6, 116.2, 121.0, 121.3, 130.0, 131.2, 161.9, 166.8.

### Refinement

All hydrogen atoms were discernible in difference Fourier maps and could be refined to reasonable geometry. According to common practice H atoms bonded to C atoms were kept in ideal positions with C–H distance 0.96 Å during the refinement. The methyl H atoms were allowed to rotate freely about the adjacent C—C bonds. The hydroxyl H atoms were found in difference Fourier maps and their coordinates were refined freely. All H atoms were refined with displacement coefficients *U*_iso_(H) set to 1.5Ueq(C, O) for methyl and hydroxyl groups and to to 1.2Ueq(C) for the CH– and CH_2_– groups.

### Crystal data

**Table:**

C_25_H_30_N_2_O_6_	*F*(000) = 1936
*M**_r_* = 454.5	*D*_x_ = 1.352 Mg m^−^^3^
Monoclinic, *C*2/*c*	Cu *K*α radiation, λ = 1.5418 Å
Hall symbol: -C 2yc	Cell parameters from 9151 reflections
*a* = 26.3472 (6) Å	θ = 3.4–67.1°
*b* = 9.1432 (1) Å	µ = 0.80 mm^−^^1^
*c* = 21.6585 (4) Å	*T* = 120 K
β = 121.139 (3)°	Block, colourless
*V* = 4465.7 (2) Å^3^	0.41 × 0.23 × 0.16 mm
*Z* = 8	

### Data collection

**Table:**

Agilent Xcalibur diffractometer with an Atlas (Gemini ultra Cu) detector	3970 independent reflections
Radiation source: Enhance Ultra (Cu) X-ray Source	3309 reflections with *I* > 3σ(*I*)
mirror	*R*_int_ = 0.028
Detector resolution: 10.3784 pixels mm^-1^	θ_max_ = 67.1°, θ_min_ = 3.9°
Rotation method data acquisition using ω scans	*h* = −31→30
Absorption correction: multi-scan (*CrysAlis PRO*; Agilent, 2010)	*k* = −10→10
*T*_min_ = 0.853, *T*_max_ = 1	*l* = −24→25
17421 measured reflections	

### Refinement

**Table:**

Refinement on *F*^2^	0 constraints
*R*[*F*^2^ > 2σ(*F*^2^)] = 0.035	H atoms treated by a mixture of independent and constrained refinement
*wR*(*F*^2^) = 0.097	Weighting scheme based on measured s.u.'s *w* = 1/[σ^2^(*I*) + 0.0016*I*^2^]
*S* = 1.54	(Δ/σ)_max_ = 0.045
3970 reflections	Δρ_max_ = 0.26 e Å^−^^3^
304 parameters	Δρ_min_ = −0.18 e Å^−^^3^
0 restraints	

### Special details

**Table:**

Experimental. CrysAlisPro (Agilent Technologies, 2010) Empirical absorption correction using spherical harmonics, implemented in SCALE3 ABSPACK scaling algorithm.
Refinement. The refinement was carried out against all reflections. The conventional *R*-factor is always based on *F*. The goodness of fit as well as the weighted *R*-factor are based on *F* and *F*^2^ for refinement carried out on *F* and *F*^2^, respectively. The threshold expression is used only for calculating *R*-factors *etc*. and it is not relevant to the choice of reflections for refinement.The program used for refinement, Jana2006, uses the weighting scheme based on the experimental expectations, see _refine_ls_weighting_details, that does not force *S* to be one. Therefore the values of *S* are usually larger than the ones from the *SHELX* program.

### Fractional atomic coordinates and isotropic or equivalent isotropic displacement parameters (Å^2^)

**Table:**

	*x*	*y*	*z*	*U*_iso_*/*U*_eq_	
O1	0.25904 (4)	0.33029 (11)	1.07162 (5)	0.0309 (4)	
O2	0.18467 (4)	0.18439 (10)	1.05612 (5)	0.0305 (4)	
O3	0.03275 (4)	0.68882 (11)	0.84111 (5)	0.0269 (4)	
O4	0.01966 (4)	0.25644 (10)	0.51016 (5)	0.0275 (4)	
O5	0.10151 (4)	0.15258 (9)	0.52210 (5)	0.0246 (4)	
O6	0.22213 (4)	0.68869 (11)	0.72919 (5)	0.0281 (4)	
N1	0.11660 (5)	0.80792 (11)	0.82539 (6)	0.0206 (4)	
N2	0.12619 (5)	0.80593 (11)	0.72269 (6)	0.0203 (4)	
C1	0.12226 (6)	0.70921 (14)	0.77488 (7)	0.0229 (5)	
C2	0.12528 (6)	0.95764 (13)	0.80739 (7)	0.0200 (5)	
C3	0.09573 (6)	1.07952 (14)	0.82484 (7)	0.0244 (6)	
C4	0.10153 (7)	1.22240 (15)	0.79175 (8)	0.0282 (6)	
C5	0.07990 (6)	1.20809 (15)	0.71143 (8)	0.0283 (6)	
C6	0.10988 (6)	1.08124 (14)	0.69607 (7)	0.0252 (6)	
C7	0.09940 (6)	0.94404 (13)	0.72716 (7)	0.0203 (5)	
C8	0.15534 (6)	0.76766 (14)	0.90155 (7)	0.0221 (5)	
C9	0.13584 (6)	0.62646 (14)	0.91905 (6)	0.0201 (5)	
C10	0.17667 (6)	0.52797 (14)	0.96778 (7)	0.0209 (5)	
C11	0.15941 (6)	0.40115 (14)	0.98767 (7)	0.0217 (5)	
C12	0.09912 (6)	0.37225 (14)	0.95701 (7)	0.0232 (6)	
C13	0.05745 (6)	0.46807 (14)	0.90719 (7)	0.0234 (6)	
C14	0.07510 (6)	0.59530 (14)	0.88841 (7)	0.0208 (5)	
C15	0.20657 (6)	0.30532 (14)	1.04216 (7)	0.0238 (6)	
C16	0.22831 (7)	0.08821 (16)	1.11053 (9)	0.0354 (7)	
C17	0.09522 (6)	0.74531 (14)	0.64881 (7)	0.0223 (5)	
C18	0.12328 (5)	0.60631 (14)	0.64261 (7)	0.0196 (5)	
C19	0.08850 (6)	0.49718 (13)	0.59512 (7)	0.0193 (5)	
C20	0.11324 (6)	0.37195 (13)	0.58482 (7)	0.0202 (5)	
C21	0.17476 (6)	0.35599 (14)	0.62305 (7)	0.0227 (5)	
C22	0.21021 (6)	0.46283 (14)	0.67116 (7)	0.0243 (5)	
C23	0.18496 (6)	0.58707 (14)	0.68127 (7)	0.0216 (5)	
C24	0.07276 (6)	0.25842 (14)	0.53542 (7)	0.0209 (5)	
C25	0.06444 (6)	0.03656 (14)	0.47537 (8)	0.0280 (6)	
H1a	0.087447	0.649231	0.749828	0.0275*	
H1b	0.158079	0.653125	0.801001	0.0275*	
H2	0.166122	0.986613	0.835672	0.024*	
H3a	0.114963	1.0903	0.876215	0.0293*	
H3b	0.05453	1.05683	0.804644	0.0293*	
H4a	0.079681	1.2984	0.798436	0.0338*	
H4b	0.142166	1.253728	0.817484	0.0338*	
H5a	0.037711	1.194144	0.684606	0.034*	
H5b	0.087307	1.297828	0.694408	0.034*	
H6a	0.092156	1.070066	0.644902	0.0302*	
H6b	0.151703	1.09954	0.719443	0.0302*	
H7	0.056997	0.938134	0.697421	0.0244*	
H8a	0.155072	0.844511	0.931581	0.0265*	
H8b	0.195371	0.757804	0.912249	0.0265*	
H10	0.218184	0.547519	0.988554	0.0251*	
H12	0.08652	0.285732	0.970499	0.0278*	
H13	0.01599	0.44652	0.885411	0.0281*	
H16a	0.20887	0.004224	1.115555	0.0531*	
H16b	0.255737	0.057284	1.096498	0.0531*	
H16c	0.249366	0.139159	1.155739	0.0531*	
H17a	0.094472	0.817116	0.616009	0.0268*	
H17b	0.054477	0.727007	0.633433	0.0268*	
H19	0.046172	0.508256	0.568587	0.0232*	
H21	0.192427	0.270804	0.615918	0.0272*	
H22	0.25252	0.451248	0.697763	0.0291*	
H25a	0.088144	−0.030455	0.466731	0.0421*	
H25b	0.046388	−0.014333	0.49793	0.0421*	
H25c	0.034108	0.077264	0.430369	0.0421*	
H3	0.0529 (8)	0.750 (2)	0.8286 (10)	0.0404*	
H6	0.1986 (8)	0.751 (2)	0.7320 (10)	0.0422*	

### Atomic displacement parameters (Å^2^)

**Table:**

	*U*^11^	*U*^22^	*U*^33^	*U*^12^	*U*^13^	*U*^23^
O1	0.0282 (5)	0.0290 (5)	0.0291 (5)	0.0015 (4)	0.0104 (4)	0.0040 (4)
O2	0.0339 (5)	0.0213 (5)	0.0341 (5)	0.0023 (4)	0.0159 (5)	0.0076 (4)
O3	0.0212 (5)	0.0296 (5)	0.0295 (5)	0.0038 (4)	0.0127 (4)	0.0069 (4)
O4	0.0223 (5)	0.0260 (5)	0.0278 (5)	0.0000 (4)	0.0085 (4)	−0.0035 (4)
O5	0.0274 (5)	0.0199 (5)	0.0270 (5)	−0.0007 (4)	0.0145 (4)	−0.0035 (4)
O6	0.0228 (5)	0.0260 (5)	0.0319 (5)	−0.0039 (4)	0.0116 (4)	−0.0081 (4)
N1	0.0240 (5)	0.0190 (5)	0.0183 (5)	−0.0006 (4)	0.0107 (5)	0.0009 (4)
N2	0.0236 (5)	0.0187 (5)	0.0180 (5)	0.0017 (4)	0.0103 (4)	0.0004 (4)
C1	0.0280 (7)	0.0202 (6)	0.0213 (6)	0.0012 (5)	0.0131 (6)	0.0010 (5)
C2	0.0209 (6)	0.0179 (6)	0.0209 (6)	−0.0006 (5)	0.0106 (5)	0.0013 (5)
C3	0.0281 (7)	0.0239 (7)	0.0228 (7)	0.0030 (5)	0.0144 (6)	0.0004 (5)
C4	0.0368 (8)	0.0201 (7)	0.0310 (8)	0.0052 (6)	0.0199 (7)	0.0015 (6)
C5	0.0344 (8)	0.0219 (7)	0.0297 (7)	0.0059 (6)	0.0173 (6)	0.0062 (6)
C6	0.0327 (7)	0.0226 (7)	0.0222 (7)	0.0031 (6)	0.0156 (6)	0.0032 (5)
C7	0.0202 (6)	0.0199 (6)	0.0204 (6)	0.0027 (5)	0.0101 (5)	0.0008 (5)
C8	0.0225 (6)	0.0233 (6)	0.0182 (6)	−0.0015 (5)	0.0089 (5)	0.0009 (5)
C9	0.0229 (6)	0.0219 (6)	0.0168 (6)	−0.0020 (5)	0.0111 (5)	−0.0024 (5)
C10	0.0218 (6)	0.0234 (7)	0.0174 (6)	−0.0015 (5)	0.0099 (5)	−0.0028 (5)
C11	0.0262 (7)	0.0198 (6)	0.0201 (6)	−0.0008 (5)	0.0127 (6)	−0.0031 (5)
C12	0.0294 (7)	0.0191 (6)	0.0253 (7)	−0.0019 (5)	0.0171 (6)	−0.0028 (5)
C13	0.0228 (7)	0.0248 (7)	0.0251 (7)	−0.0021 (5)	0.0141 (6)	−0.0035 (5)
C14	0.0215 (6)	0.0232 (6)	0.0182 (6)	0.0026 (5)	0.0106 (5)	−0.0010 (5)
C15	0.0294 (7)	0.0210 (7)	0.0217 (6)	−0.0002 (5)	0.0136 (6)	−0.0024 (5)
C16	0.0412 (8)	0.0257 (7)	0.0366 (8)	0.0081 (6)	0.0183 (7)	0.0110 (6)
C17	0.0244 (7)	0.0228 (7)	0.0168 (6)	0.0033 (5)	0.0087 (5)	0.0006 (5)
C18	0.0230 (6)	0.0204 (6)	0.0164 (6)	0.0018 (5)	0.0109 (5)	0.0026 (5)
C19	0.0191 (6)	0.0227 (6)	0.0162 (6)	0.0015 (5)	0.0092 (5)	0.0040 (5)
C20	0.0236 (6)	0.0202 (6)	0.0180 (6)	−0.0003 (5)	0.0116 (5)	0.0035 (5)
C21	0.0242 (7)	0.0194 (6)	0.0255 (7)	0.0027 (5)	0.0137 (6)	0.0024 (5)
C22	0.0193 (6)	0.0245 (7)	0.0274 (7)	0.0011 (5)	0.0109 (6)	0.0022 (5)
C23	0.0234 (6)	0.0221 (6)	0.0190 (6)	−0.0035 (5)	0.0108 (5)	−0.0001 (5)
C24	0.0258 (7)	0.0200 (6)	0.0165 (6)	0.0030 (5)	0.0106 (5)	0.0040 (5)
C25	0.0338 (7)	0.0207 (7)	0.0276 (7)	−0.0019 (6)	0.0145 (6)	−0.0046 (5)

### Geometric parameters (Å, °)

**Table:**

O1—C15	1.2075 (17)	C7—H7	0.96
O2—C15	1.3519 (19)	C8—C9	1.509 (2)
O2—C16	1.4432 (16)	C8—H8a	0.96
O3—C14	1.3578 (14)	C8—H8b	0.96
O3—H3	0.90 (2)	C9—C10	1.3812 (16)
O4—C24	1.2105 (17)	C9—C14	1.4088 (19)
O5—C24	1.3488 (19)	C10—C11	1.393 (2)
O5—C25	1.4426 (15)	C10—H10	0.96
O6—C23	1.3595 (14)	C11—C12	1.394 (2)
O6—H6	0.87 (2)	C11—C15	1.4791 (16)
N1—C1	1.484 (2)	C12—C13	1.3828 (16)
N1—C2	1.4732 (17)	C12—H12	0.96
N1—C8	1.4686 (15)	C13—C14	1.389 (2)
N2—C1	1.480 (2)	C13—H13	0.96
N2—C7	1.4741 (18)	C16—H16a	0.96
N2—C17	1.4775 (16)	C16—H16b	0.96
C1—H1a	0.96	C16—H16c	0.96
C1—H1b	0.96	C17—C18	1.511 (2)
C2—C3	1.515 (2)	C17—H17a	0.96
C2—C7	1.5081 (19)	C17—H17b	0.96
C2—H2	0.96	C18—C19	1.3850 (16)
C3—C4	1.535 (2)	C18—C23	1.4021 (18)
C3—H3a	0.96	C19—C20	1.391 (2)
C3—H3b	0.96	C19—H19	0.96
C4—C5	1.531 (2)	C20—C21	1.3951 (18)
C4—H4a	0.96	C20—C24	1.4763 (16)
C4—H4b	0.96	C21—C22	1.3804 (17)
C5—C6	1.533 (2)	C21—H21	0.96
C5—H5a	0.96	C22—C23	1.390 (2)
C5—H5b	0.96	C22—H22	0.96
C6—C7	1.515 (2)	C25—H25a	0.96
C6—H6a	0.96	C25—H25b	0.96
C6—H6b	0.96	C25—H25c	0.96
			
C15—O2—C16	115.40 (11)	C8—C9—C14	120.41 (10)
C14—O3—H3	103.5 (11)	C10—C9—C14	118.31 (13)
C24—O5—C25	115.23 (11)	C9—C10—C11	121.95 (13)
C23—O6—H6	104.1 (11)	C9—C10—H10	119.0222
C1—N1—C2	106.36 (12)	C11—C10—H10	119.0236
C1—N1—C8	113.53 (10)	C10—C11—C12	118.98 (11)
C2—N1—C8	114.66 (9)	C10—C11—C15	117.78 (12)
C1—N2—C7	103.68 (13)	C12—C11—C15	123.23 (13)
C1—N2—C17	112.66 (10)	C11—C12—C13	120.07 (14)
C7—N2—C17	112.18 (9)	C11—C12—H12	119.9668
N1—C1—N2	105.84 (10)	C13—C12—H12	119.9653
N1—C1—H1a	109.4722	C12—C13—C14	120.53 (13)
N1—C1—H1b	109.4722	C12—C13—H13	119.7345
N2—C1—H1a	109.4704	C14—C13—H13	119.7351
N2—C1—H1b	109.4705	O3—C14—C9	121.21 (12)
H1a—C1—H1b	112.8746	O3—C14—C13	118.64 (12)
N1—C2—C3	116.58 (14)	C9—C14—C13	120.14 (11)
N1—C2—C7	100.87 (10)	O1—C15—O2	122.75 (11)
N1—C2—H2	111.3309	O1—C15—C11	124.71 (14)
C3—C2—C7	111.32 (10)	O2—C15—C11	112.54 (12)
C3—C2—H2	100.891	O2—C16—H16a	109.472
C7—C2—H2	116.5959	O2—C16—H16b	109.4708
C2—C3—C4	108.75 (15)	O2—C16—H16c	109.4708
C2—C3—H3a	109.472	H16a—C16—H16b	109.4716
C2—C3—H3b	109.4708	H16a—C16—H16c	109.4711
C4—C3—H3a	109.4717	H16b—C16—H16c	109.471
C4—C3—H3b	109.471	N2—C17—C18	112.97 (9)
H3a—C3—H3b	110.1798	N2—C17—H17a	109.4721
C3—C4—C5	112.91 (11)	N2—C17—H17b	109.469
C3—C4—H4a	109.4727	C18—C17—H17a	109.4721
C3—C4—H4b	109.4709	C18—C17—H17b	109.471
C5—C4—H4a	109.4714	H17a—C17—H17b	105.728
C5—C4—H4b	109.4703	C17—C18—C19	120.39 (11)
H4a—C4—H4b	105.7947	C17—C18—C23	121.45 (10)
C4—C5—C6	112.36 (11)	C19—C18—C23	118.06 (13)
C4—C5—H5a	109.4713	C18—C19—C20	121.81 (12)
C4—C5—H5b	109.4717	C18—C19—H19	119.0941
C6—C5—H5a	109.4708	C20—C19—H19	119.0943
C6—C5—H5b	109.4712	C19—C20—C21	119.18 (11)
H5a—C5—H5b	106.4244	C19—C20—C24	118.18 (12)
C5—C6—C7	107.28 (15)	C21—C20—C24	122.60 (13)
C5—C6—H6a	109.4711	C20—C21—C22	119.93 (13)
C5—C6—H6b	109.4712	C20—C21—H21	120.0335
C7—C6—H6a	109.4714	C22—C21—H21	120.0348
C7—C6—H6b	109.4712	C21—C22—C23	120.34 (12)
H6a—C6—H6b	111.5736	C21—C22—H22	119.8287
N2—C7—C2	101.53 (9)	C23—C22—H22	119.8289
N2—C7—C6	118.45 (15)	O6—C23—C18	121.67 (13)
N2—C7—H7	109.7793	O6—C23—C22	117.68 (11)
C2—C7—C6	111.47 (10)	C18—C23—C22	120.66 (11)
C2—C7—H7	116.9848	O4—C24—O5	122.57 (11)
C6—C7—H7	99.4758	O4—C24—C20	124.81 (14)
N1—C8—C9	111.57 (9)	O5—C24—C20	112.61 (12)
N1—C8—H8a	109.4706	O5—C25—H25a	109.4719
N1—C8—H8b	109.4706	O5—C25—H25b	109.4705
C9—C8—H8a	109.4711	O5—C25—H25c	109.4708
C9—C8—H8b	109.4718	H25a—C25—H25b	109.4709
H8a—C8—H8b	107.2874	H25a—C25—H25c	109.472
C8—C9—C10	121.21 (12)	H25b—C25—H25c	109.4712
			
C10—C11—C15—O1	1.3 (2)	C19—C20—C24—O4	−10.1 (2)

### Hydrogen-bond geometry (Å, °)

**Table:**

*D*—H···*A*	*D*—H	H···*A*	*D*···*A*	*D*—H···*A*
O3—H3···N1	0.90 (2)	1.80 (2)	2.6383 (18)	154.3 (16)
O6—H6···N2	0.87 (2)	1.88 (2)	2.6814 (18)	153.0 (18)
C2—H2···O1^i^	0.96	2.57	3.414 (2)	146
C4—H4b···O1^i^	0.96	2.58	3.353 (2)	137
C16—H16c···O6^ii^	0.96	2.59	3.350 (2)	136

Symmetry codes: (i) −*x*+1/2, −*y*+3/2, −*z*+2; (ii) *x*, −*y*+1, *z*+1/2.
